# Integrative analysis of high-throughput RNAi screen data identifies the FER and CRKL tyrosine kinases as new regulators of the mitogenic ERK-dependent pathways in transformed cells

**DOI:** 10.1186/1471-2164-15-1169

**Published:** 2014-12-23

**Authors:** Philippe Nizard, Frédéric Ezan, Dominique Bonnier, Nolwenn Le Meur, Sophie Langouët, Georges Baffet, Yannick Arlot-Bonnemains, Nathalie Théret

**Affiliations:** CNRS UMR6290, Institut de Génétique et développement de Rennes IGDR, Université Rennes 1, SFR Biosit, Rennes, France; UMRS 1147-Medecine Personnalisée, Pharmacogénomique et Optimisation Thérapeutique, Université Paris Descartes, Centre Universitaire des Saints-Pères, Paris, France; INSERM UMR1085, Institut de Recherche sur la Santé l’Environnement et le Travail IRSET, Université Rennes 1, SFR Biosit, Rennes, France; EHESP Rennes, Sorbonne Paris Cité, équipe Management des organisations en santé (MOS), Paris, France

**Keywords:** Kinases, Integrative genomics, Cell proliferation

## Abstract

**Background:**

Cell proliferation is a hallmark of cancer and depends on complex signaling networks that are chiefly supported by protein kinase activities. Therapeutic strategies have been used to target specific kinases but new methods are required to identify combined targets and improve treatment. Here, we propose a small interfering RNA genetic screen and an integrative approach to identify kinase networks involved in the proliferation of cancer cells.

**Results:**

The functional siRNA screen of 714 kinases in HeLa cells identified 91 kinases implicated in the regulation of cell growth, most of them never being reported in previous whole-genome siRNA screens. Based on gene ontology annotations, we have further discriminated between two classes of kinases that, when suppressed, result in alterations of the mitotic index and provoke cell-cycle arrest. Extinguished kinases that lead to a low mitotic index mostly include kinases implicated in cytosolic signaling. In contrast, extinguished kinases that result in a high mitotic index mostly include kinases implicated in cell division. By mapping hit kinases in the PhosphPOINT phosphoprotein database, we generated scale-free networks consisting of 449 and 661 protein-protein interactions for kinases from low MI and high MI groups, respectively. Further analyses of the kinase interactomes revealed specific modules such as FER- and CRKL-containing modules that connect three members of the epidermal growth factor receptor (EGFR) family, suggesting a tight control of the mitogenic EGF-dependent pathway. Based on experimental studies, we confirm the involvement of these two kinases in the regulation of tumor cell growth.

**Conclusion:**

Based on a combined approach of large kinome-wide siRNA screens and ontology annotations, our study identifies for the first time two kinase groups differentially implicated in the control of cell proliferation. We further demonstrate that integrative analysis of the kinase interactome provides key information which can be used to facilitate or optimize target design for new therapeutic strategies. The complete list of protein-protein interactions from the two functional kinase groups will provide a useful database for future investigations.

**Electronic supplementary material:**

The online version of this article (doi:10.1186/1471-2164-15-1169) contains supplementary material, which is available to authorized users.

## Background

The protein kinase family is one of the largest gene families in the human genome and protein phosphorylation affects more than 30% of all proteins. Most kinases are involved in signal transduction pathways that govern cell proliferation, differentiation and apoptosis. Protein kinase expression and activities are highly misregulated in cancer, justifying the development of therapeutic strategies that target kinases. Inhibitors of protein kinase oncogenes such as gefinitib for EGFR [1], imatinib for BCR-ABL [2] or trastuzumab for HER2 [3] have been subjected to clinical assays, but the efficiency of targeting specific kinase oncogenes has been impaired by the intrinsic heterogeneity of cancers. In order to improve antitumor treatment, investigation of the non-oncogene dependency of cancer, combined therapies and multiple-target approaches have been proposed [4–7]. These have proven to be highly complex tasks, and an integrated vision of kinome networks is required to optimize for the best combinations of targets. Over the past decade, high-throughput approaches have significantly contributed to the global picture of kinase profiles in cancer and cell proliferation, mainly by describing the differential expression and activation of numerous kinases. However, the basis for the dynamic complexity of kinase networks remains unclear. Unlike global analyses such as gene-expression array and proteomics, RNA interference (RNAi) technology is a functional approach that has been used both to identify new selective targets and to understand the cell’s response to cancer drugs [8]. Using single-well screening, the small interference RNA (siRNA) method was shown to be more suitable for phenotypic analysis and has been successfully employed to investigate genes involved in the cell cycle and in cell proliferation [9–11]. Taken together, genome-wide RNAi screens have led to the identification of more than 2 500 genes that are implicated in cell proliferation but basing the rational choice of efficient targets on these data has proven difficult. More recently, integrative screening combining genome-wide RNAi screens with multiple biologic data have been developed to filter for high-confidence candidate targets [12]. However combining results from different resources to extract information of interest remains a challenging task. The originality of the work reported here consists in the specific screen of a set of 714 kinases and, using integrative data-mining analyses to filter functional kinase groups, constructing kinase interaction networks that successfully identify new biologically relevant targets.

For this purpose, we developed an image-based RNAi screen to identify kinases required for cell-cycle progression. The readout of the screen consists in the quantification of mitotic index (phospho-histone H3-positive cells) after RNAi treatment. Based on ontology annotations, two groups of kinases leading to either low or high mitotic index (MI) were functionally characterized. By extracting information from PhosphoPOINT, the human interactome and phosphoprotein database, we further generated protein networks that permit the identification of two key kinases as regulators of tumor cell growth that control cell growth, the FER and CRKL tyrosine kinases that form a pivotal subnetwork which controls the EGFR mitogenic pathway.

## Results and discussion

**Figure 1:**
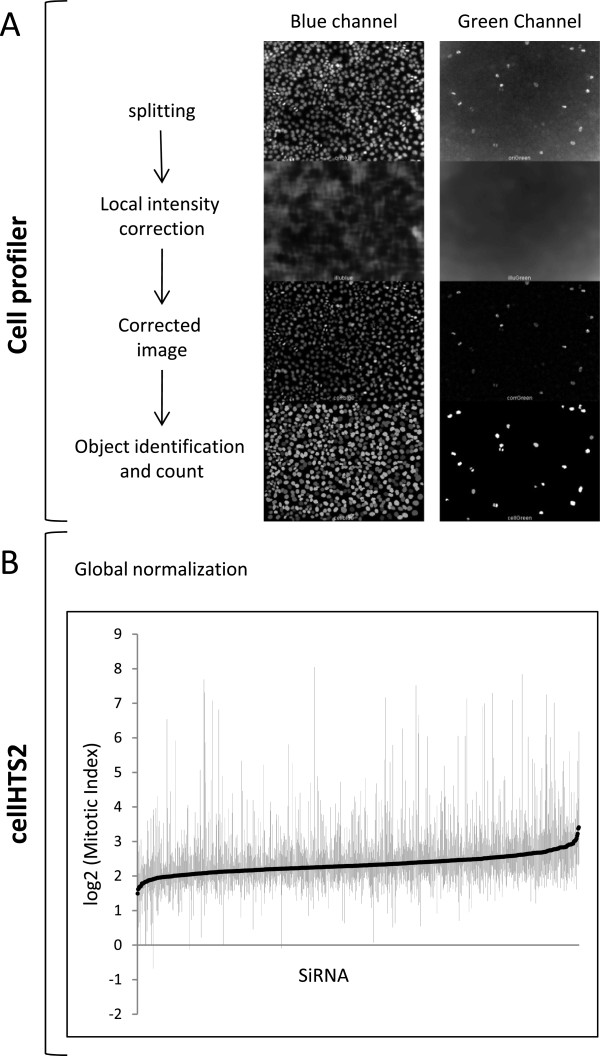
**Experimental workflow to identify kinase targets using high-content imaging. (A)** Cells were fluorescently labeled for DNA (DAPI staining, blue channel) and for phosphorylated histone H3 (green channel) as indicated above the photographs. Using CellProfiler software, snapshots were split into blue and green channels. For each image a local correction was applied and objects (nucleus and phosphohistone H3-positive cells) were counted. **(B)** Normalization and Mitotic Index quantification were performed using CellHTS2 free software. The diagram depicts box-plots of the whole experiment.

**Figure 2:**
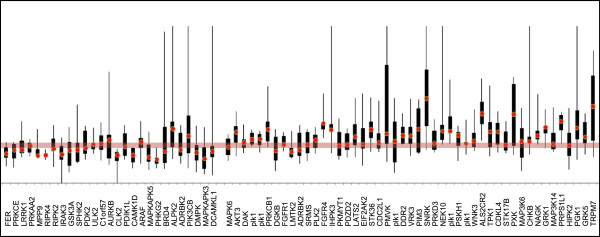
**Variation of the mitotic index (MI) in HeLa cells transfected with siRNA targeted against kinases.** Illustration of MI variation of kinases for which siRNA significantly induced either an increase (High) or a decrease (Low) in mitotic index compared to the mean MI of cycling cells reported as 5% in the literature (red band).

Protein kinases control the cell cycle and their deregulation has been widely reported in cancers. As a consequence, targeted inhibition of kinases in cancer therapy has been extensively studied but identifying combinations of protein kinase targets is required to improve therapeutic strategies. RNAi strategies based on genome-wide screens have been previously used to identify genes involved in the cell cycle and mitosis, but with a low redundancy of identified kinase genes [9–18]. Less than five kinases have been recovered in at least two studies, with Plk1 as the only common hit. In the present study, we have focused on genes coding for kinases by screening a siRNA library targeting 714 kinases (Additional file 1: Table S1). We then developed a robust RNAi assay to identify modifications in HeLa cell proliferation and mitosis. Cells were transfected with 3 individual siRNA duplexes targeting each kinase and cultured for 48 hours. Next, cells were fluorescently labeled for DNA (DAPI staining) and phosphorylated histone H3 (pH3), a marker for mitotic cells (Figure 1). The mitotic index was calculated as the ratio between the number of DAPI-stained cells and pH3-positive cells (Additional file 1: Table S2). On a log2 scale, values above or below the median ± 2 median absolute deviations (MAD) were selected as primary hits. As illustrated in Figure 2 and listed in Table 1, 91 primary hits led to significant variations of the mitotic index (MI) when compared to the mean MI of cycling cells (5%). From this analysis, we could identify two groups of kinases whose inhibition leads to low (n = 41) and high MI (n = 50). Comparisons with data from the literature show that 28 kinases had been already identified, as well as 13 additional related kinases (Additional file 1: Table S3). Note that results from this analysis included data from 8 published studies based on whole-genome RNAi screens, suggesting that use of dedicated siRNA libraries greatly improves the identification of kinases that interfere with cell proliferation.

**Table 1:** **Lists of protein kinases whose inhibition leads to low and high Mitotic Index**

Low mitotic index		High mitotic index	
Symbol	Gene description	Symbol	Gene description
ACVR1B	Activin A receptor, type IB	AKT3	V-akt murine thymoma viral oncogene homolog 3
ADRBK2	Adrenergic, beta, receptor kinase 2	ALS2CR2	Amyotrophic lateral sclerosis 2 (juvenile) chromosome region
AK2	Adenylate kinase 2	BUB1	BUB1 budding uninhibited by benzimidazoles 1 homolog (yeast)
ALPK2	Alpha-kinase 2	CAMK1	Calcium/calmodulin-dependent protein kinase I
ARAF	V-raf murine sarcoma 3611 viral oncogene homolog	CDC2L1	Cell division cycle 2-like protein kinase 1
AURKB	Aurora kinase B	CDC7	Cell division cycle 7 homolog (S. cerevisiae)
BRD4	Bromodomain containing 4	CDKL4	Cyclin-dependent kinase-like 4
BTK	Bruton agammaglobulinemia tyrosine kinase	CHKB	choline kinase beta
CAMK1D	Calcium/calmodulin-dependent protein kinase ID	CIT	Citron (rho-interacting, serine/threonine kinase 21)
CDC42BPG	CDC42 binding protein kinase gamma (DMPK-like)	DAK	Dihydroxyacetone kinase 2 homolog (S. cerevisiae)
CDKL3	Cyclin-dependent kinase-like 3	DDR2	Discoidin domain receptor family, member 2
CLK2	CDC-like kinase 2	DGKB	Diacylglycerol kinase, beta 90 kDa
CRKL	V-crk sarcoma virus CT10 oncogene homolog (avian)-like	EIF2AK2	Eukaryotic translation initiation factor 2-alpha kinase 2
DCAMKL1	Doublecortin-like and CAM kinase-like 1	ETNK2	Ethanolamine kinase 2
DGKE	Diacylglycerol kinase, epsilon 64 kDa	FGFR1	Fibroblast growth factor receptor 1
DMPK	Dystrophia myotonica-protein kinase	FGFR4	Fibroblast growth factor receptor 4
FER	Fer (fps/fes related) tyrosine kinase	GRK1	G protein-coupled receptor kinase 1
FGFR3	Fibroblast growth factor receptor 3	GRK5	G protein-coupled receptor kinase 5
GSK3A	Glycogen synthase kinase 3 alpha	HIPK2	Homeodomain interacting protein kinase 2
IRAK3	Interleukin-1 receptor-associated kinase 3	IGF1R	Insulin-like growth factor 1 receptor
LMTK2	Lemur tyrosine kinase 2	IHPK3	Inositol hexaphosphate kinase 3
LRRK1	Leucine-rich repeat kinase 1	LATS2	LATS, large tumor suppressor, homolog 2 (Drosophila)
MAPKAPK3	Mitogen-activated protein kinase-activated protein kinase 3	MAP3K14	Mitogen-activated protein kinase kinase kinase 14
MAPKAPK5	Mitogen-activated protein kinase-activated protein kinase 5	MAP3K6	Mitogen-activated protein kinase kinase kinase 6
MARK1	MAP/microtubule affinity-regulating kinase 1	MAPK6	Mitogen-activated protein kinase 6
MPP3	Membrane protein, palmitoylated 3	NAGK	N-acetylglucosamine kinase
PDIK1L	PDLIM1 interacting kinase 1 like	NEK10	NIMA (never in mitosis gene a)- related kinase 10
PDK2	Pyruvate dehydrogenase kinase, isozyme 2	PDZD2	PDZ domain containing 2
PGK2	Phosphoglycerate kinase 2	PGK1	Phosphoglycerate kinase 1
PHKA1	Phosphorylase kinase, alpha 1 (muscle)	PIM3	Pim-3 oncogene
PHKG2	Phosphorylase kinase, gamma 2 (testis)	PKMYT1	Protein kinase, membrane associated tyrosine/threonine 1
PIK3CB	Phosphoinositide-3-kinase, catalytic, beta polypeptide	PLK1	Polo-like kinase 1 (Drosophila)
PRKAA2	Protein kinase, AMP-activated, alpha 2 catalytic subunit	PLK2	Polo-like kinase 2 (Drosophila)
PRKCE	Protein kinase C, epsilon	PMVK	Phosphomevalonate kinase
RIPK2	Receptor-interacting serine-threonine kinase 2	PRKAR2B	Protein kinase, cAMP-dependent, regulatory, type II, beta
RIPK4	Receptor-interacting serine-threonine kinase 4	PRKCB1	Protein kinase C, beta 1
SPHK2	sphingosine kinase 2	PRKD3	Protein kinase D3
STC1	Stanniocalcin 1	PRKDC	Protein kinase, DNA-activated, catalytic polypeptide
STK40	Serine/threonine kinase 40	PRPS1L1	Phosphoribosyl pyrophosphate synthetase 1-like 1
TNK1	Tyrosine kinase, non-receptor, 1	PSKH1	Protein serine kinase H1
ULK2	Unc-51-like kinase 2 (C. elegans)	PXK	PX domain containing serine/threonine kinase
		SNRK	SNF related kinase
		SRMS	Src-related kinase
		STK17B	Serine/threonine kinase 17b
		STK36	Serine/threonine kinase 36, fused homolog (Drosophila)
		TNIK	TRAF2 and NCK interacting kinase
		TPK1	Thiamin pyrophosphokinase 1
		TRPM7	Transient receptor potential cation channel, subfamily M, member 7
		VRK3	Vaccinia related kinase 3
		WNK3	WNK lysine deficient protein kinase 3

### Ontology annotations discriminate between functionally different kinase groups

**Figure 3:**
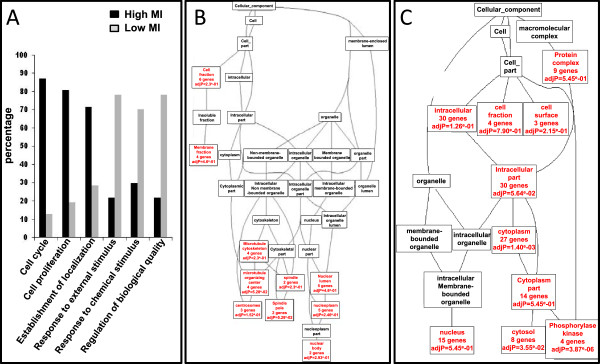
**Ontology analysis of kinases. (A)** Comparative annotations using Fatigo tool of biological processes between kinases for which siRNA induced either an increase (High) or a decrease (Low) in mitotic index (MI). **(B)** and **(C)**, Directed acyclic graphs from GOTree Machine (GOTM) describing enrichment in cellular component ontology for high-MI **(B)** and low-MI **(C)** groups (significant terms are indicated as red box including p value).

To better understand the significance of the existence of two distinct kinase groups whose knockdown leads to opposite effects, we investigated their functional annotations using Gene Ontology analysis. Based on a classical hypergeometric test for Gene Ontology term enrichment, the FATIGO tool provides a t-test for cross-comparison enrichment analyses from two gene lists. As shown in Figure 3, the two kinase lists (High and Low MI) were characterized by different biological processes. Kinases from the RNAi assay that induced a high MI are associated with the ontology terms "*cell cycle"* and "*cell proliferation*", while kinases from the RNAi assay that induced a low MI are associated with the ontology terms "*response to external and chemical stimulus"*. In accordance with these observations, we found that the two groups of kinases were also differentially enriched in cellular component terms compared with the whole-genome annotations (Figure 3B and C). Kinases for which siRNA treatment induced a high MI were enriched in nucleus-related terms that include "*microtubule organizing center"* and "*spindle pole"* (Figure 3B), while kinases for which siRNA treatment induced a low MI were enriched in cytosol-related terms, with 27 kinases annotated with the "*cytoplasm*" term (Figure 3C). Taken together, this ontology-based characterization strongly suggests that kinases from each MI group are associated with specific biologic functions.

Based on this ontology analysis, we postulated that an increased mitotic index is associated with cell-cycle arrest during mitosis, which involves kinases related to nuclear processes, while a diminished mitotic index is associated with cell-cycle blockage in early phases (G1/S/G2 phases), which involves kinases related to cytosolic signaling. Kinases implicated in the successful completion of mitosis were present in the high mitotic-index list. These include BUB1, which is involved in the spindle checkpoint function, Plk-1 and -2, critical regulators of cell mitosis and cytokinesis, and NEK10, from the NIMA gene family, which controls initiation of mitosis. We note that NIMA gene-family Neks have been previously implicated in the regulation of various aspects of the cell cycle and that Nek-10 is physically associated with Raf-1 and MEK1, formation of the three-protein complex being necessary for Nek-10-mediated MEK1 autoactivation [19, 20].

In contrast, numerous kinases from the low mitotic-index list are involved in signaling pathways. For example, PRKCE is a serine- and threonine-specific protein kinase activated by diacylglycerol, PIK3CB, is a Phosphoinositide 3-kinase, and DGKE is a Diacylglycerol kinase involved mainly in the regeneration of phosphatidylinositol (PI) from diacylglycerol in the PI cycle during signal transduction. Similarly, the MAPKAPK3 and MAPAPK5 kinases, which are activated by MAP kinases such as MAPK1/ERK, MAPK14/p38-alpha and MAPK11/p38-beta, mediate the signaling response to cellular stress and pro-inflammatory cytokines. In accordance with our data, Moffat et al. [13] reported gene targets involved in signaling pathways for which shRNAs induced a decrease in MI, such as diacylglycerol kinase (DGKG), interleukin-1 receptor-associated kinase 2 (RAK2) and glycogen synthase kinase 3 beta (GSK3B). However, it is important to note that our approach enriches the list of putative kinases involved in these processes, suggesting that dedicated siRNA libraries are more efficient than global-genome approaches to identify signaling kinase targets.

### Integrative phosphoproteomic approaches identify essential modules

**Figure 4:**
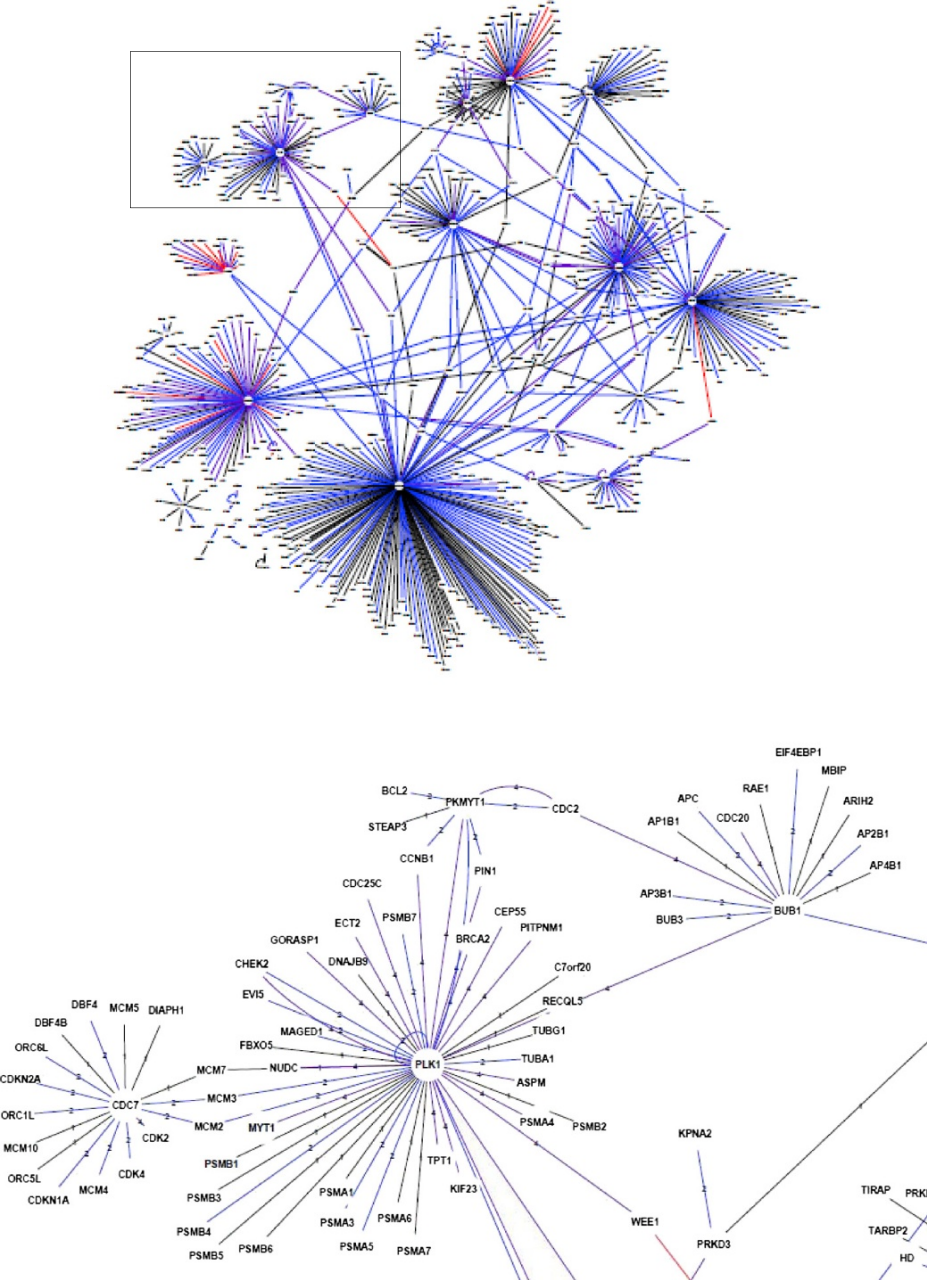
**Protein-protein interaction network for the high-MI group.** Nodes are proteins extracted from the PhosphoPOINT database using the name of kinases in the low-MI group as input. Edges represent the relationship between proteins: black edge (1) for interacting proteins; blue edges (2) for interacting proteins as well as phosphoproteins; red edges (3) for substrates and purple edges (4) for substrates as well as interacting phosphoproteins. The insert shows the mitosis regulatory module" that includes the polo-kinase 1 PLK1, the mitotic checkpoint kinase BUB1, the membrane-associated kinase PKMYT1 and the cell cycle division kinase CDC7.

**Figure 5:**
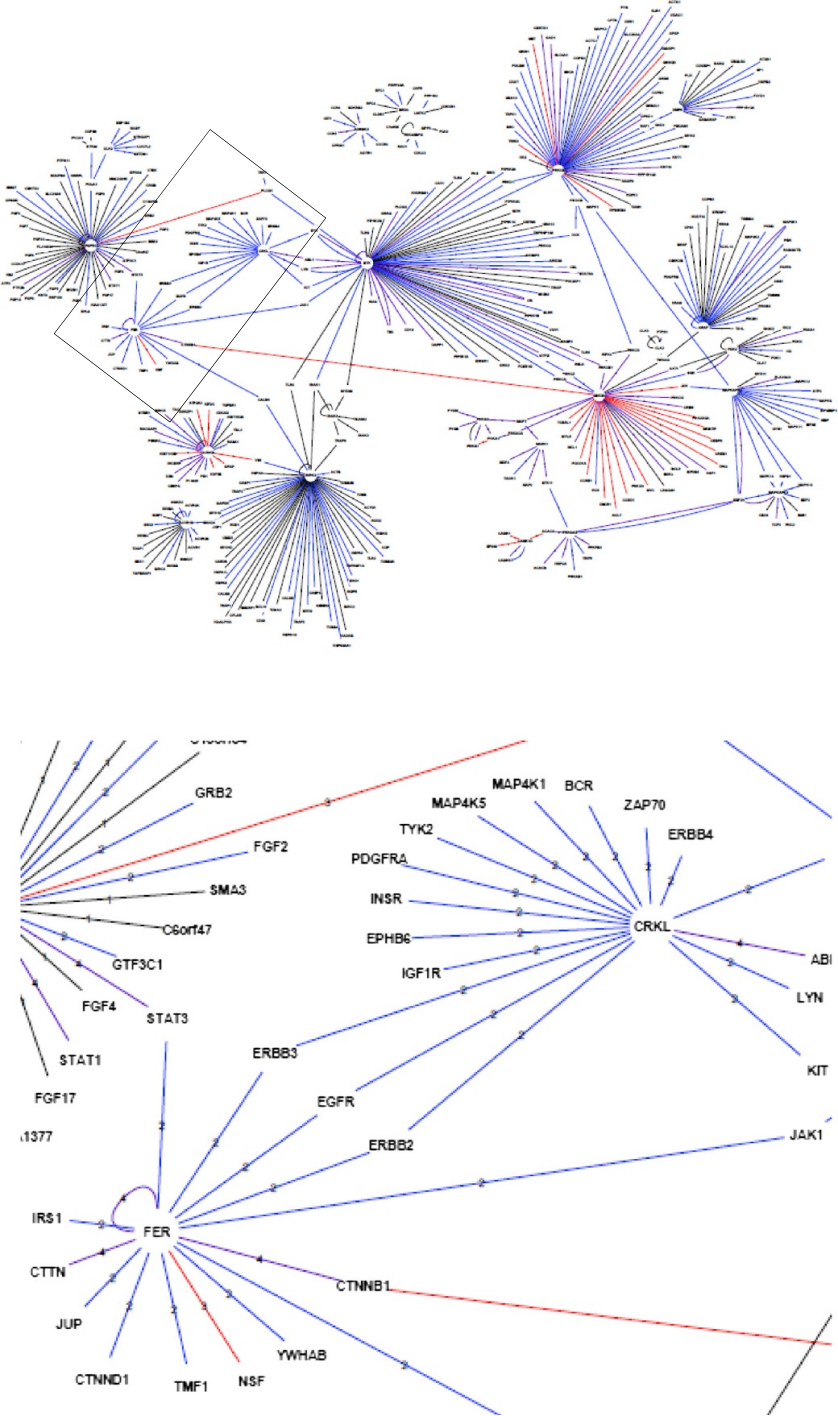
**Protein-protein interaction network for the low-MI group.** Nodes are proteins extracted from the PhosphoPOINT database using the name of kinases in the high-MI group as input. Edges represent the relationship between proteins: black edge (1) for interacting proteins; blue edges (2) for interacting proteins as well as phosphoproteins; red edges (3) for substrates and purple edges (4) for substrates as well as interacting phosphoproteins. The insert shows the EGFR sub-network connected to the FER and CRKL kinases.

**Figure 6:**
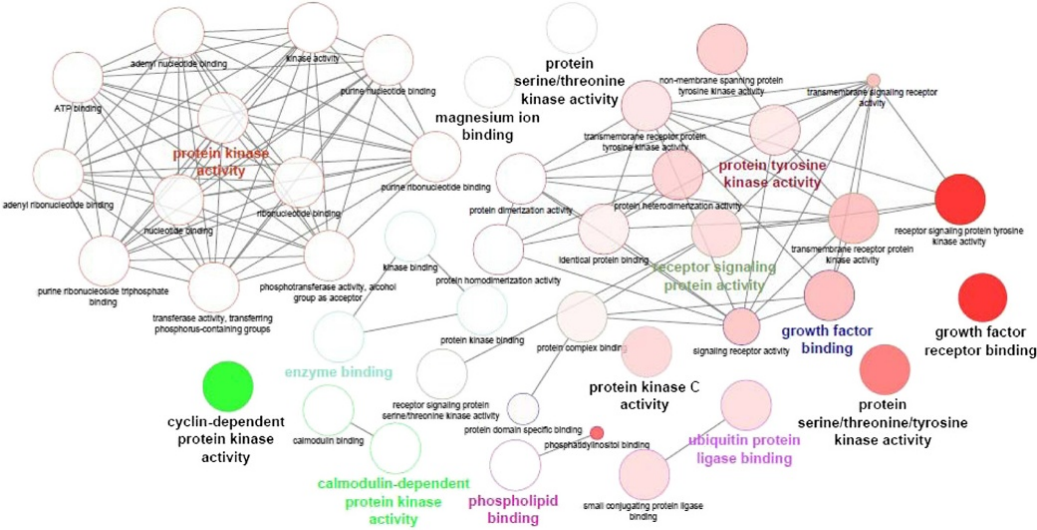
**Ontologic annotation of PPI networks.** Comparative annotation of PPIs from the low- and high-MI groups was performed using ClueGO tool as Cytoscape plug-in. Results are expressed as a graph of differential enrichment of molecular function GO terms. The color gradient shows the kinase proportion of each cluster associated with the term (green nodes for the high-MI group and red nodes for the low-MI group). Equal proportions of the two clusters are represented as white nodes.

To further understand the contribution of these signaling kinases in the control of cell proliferation, we next built the functional interactome of the two kinase groups. To do this, we first integrated protein and phosphoproteomic interactions into our data by extracting protein interactions that include phosphoprotein substrates from PhosphoPOINT, a comprehensive human kinase interactome and phospho-protein database. 449 and 661 protein-protein interactions (PPI) were identified for kinases from the low-MI and high-MI groups, respectively (detailed in Additional file 1: Tables S4 and S5). We next generated networks where nodes stand for proteins and edges represent biological information including "interacting proteins", "interacting proteins as well as phospho-proteins", "substrates" and "substrates as well as interacting phospho-proteins". As shown in Figure 4 for the kinases from the high-MI group (563 nodes) and Figure 5 for the kinases from low-MI group (406 nodes), both networks exhibit scale-free behavior, meaning that they follow a power law-degree distribution which confers scale-invariance properties and network’s robustness. Expandable views of graphs were provided as Additional file 1: Figures S1 and S2 and topology analyses were further detailed in Additional file 1: Table S6. Of note, 31/41 and 31/50 kinases from low- and high-MI groups were respectively included in PPI graphs, the remaining hit kinases not being documented in the PhosphoPOINT database. However, ontology-based comparison of the two PPI networks confirmed the differential functions of the two groups. As show in Figure 6, heat-map visualization of molecular functions showed a significant enrichment in "cyclin-dependent protein kinase activity" for PPI from the high-MI group, while PPI from the low-MI group were significantly enriched in receptor signaling-related functions such as "growth factor receptor binding", "protein serine_threonine tyrosine kinase" and "receptor signaling protein activity". These integrative PPI analyses are in agreement with the notion of specific molecular functions associated with kinases from the low-MI ("receptor signaling") and high-MI groups ("cell-cycle").

Visualization of PPI helps to identify local units of the networks, defined as modules which function as essential components of the network. Accordingly, PPI from the high-MI group showed a specific "mitosis regulatory module" that includes the polo-kinase 1 PLK1, the mitotic checkpoint kinase BUB1, the membrane-associated kinase PKMYT1 and the cell cycle division kinase CDC7 (Figure 4, insert). Note that PLK1 phosphorylates and activates BUB1 to localize it to the kinetochore, phosphorylates and inhibits the negative regulator PKMYTI and interacts with the G1/S kinase Cdc7p to target it to initiation complexes late in G1.

Focusing on signaling kinases from the low-MI group, we identified an unexpected module that includes CRKL and FER kinases and three members of the epidermal growth factor (EGF) receptor family of receptor tyrosine kinases, EGFR, ERBB2 and ERBB3 (Figure 5, insert). These interactions support a possible role of these two protein kinases as key regulators of G1/S phase progression, which is known to be driven by EGFR signaling. To validate this hypothesis, we next investigated the role of these two kinases using direct experimental approaches.

### Identification of FER and CRKL as pivotal protein kinases involved in mitogenic signaling pathways

While CRKL (CRK-like) and FER non-receptor tyrosine kinase have been previously identified using high-throughoutput screening, we demonstrate here that these two kinases work together to control the EGFR signaling pathway. Indeed, CRKL has been described as an "essential cancer-causing gene" in 12 cancer cell lines representing diverse cancer types [5]. In agreement with this observation, CRKL expression has been correlated with aggressive and malignant behavior of cancer cells, making CRKL a potential cancer marker and therapeutic target [21]. Similarly, the FER non-receptor tyrosine kinase has been previously associated with cell proliferation and cancer [22–26]. FER was initially discovered in studies focusing on the proto-oncogene Fes/Fps and was shown to play a critical role in cytoskeletal regulation, cell adhesion, migration and proliferation. FER has been associated with signaling complexes containing insulin receptor substrate-1, IGF1R and phosphatidylinositol 3-kinase [27]. Canonical IGF-IR/IRS1 signaling is activated through the binding of IRS1 to phosphorylated IGF-IR, resulting in the activation of the ERK/HIF-1α/NF-κB signaling pathway [28]. As demonstrated in Figure 5, IRS1 and IGF1R respectively interact with FER and CRKL, providing additional evidence for crosstalk between FER and CRKL in the activation of the ERK/HIF-1α/NF-κB signaling pathway.

**Figure 7:**
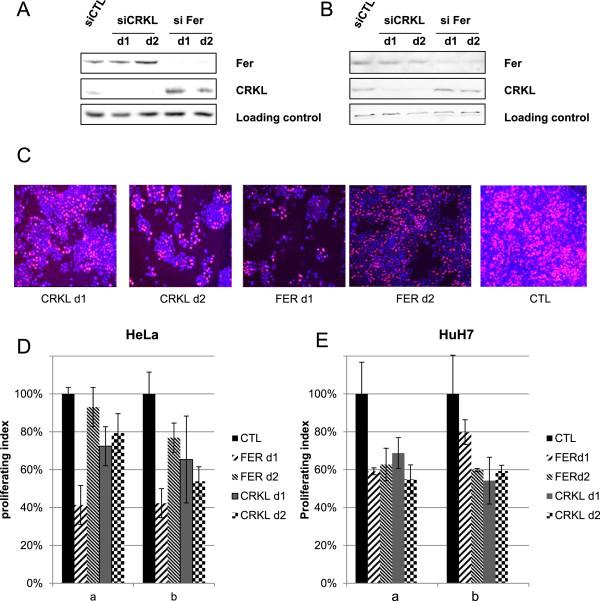
**Implication of the FER and CRKL kinases in S-phase replication of HeLa and HuH7 cells.** Cells were transfected with FER, CRKL or control siRNAs and analyzed 72 h post-transfection. **(A, B)**, western blot analysis of FER and CRKL expression in HeLa **(A)** and HuH7 **(B)**. **(C)**, Representative fluorescence microcopy images from HeLa cells (EDU labeling). **(D, E)**, Cell proliferation assay. EDU or methyl-Thymidine incorporations were performed 72 h (a) and 96 h (b) after transfections of HeLa **(D)** and HuH7 **(E)** cells. Replication is expressed as the proliferation index (EDU for HeLa cells) and cpm/μg of protein (methyl-^3^H-Thymidine for HuH7). The statistical significance was (P < 0.05) and (P < 0.001) in HeLa and HuH7 cells, respectively, relative to the control siRNA.

To validate and further investigate the effects of CRKL and FER, we analyzed cell-cycle progression in two proliferating cell lines: the cervical cancer HeLa cell line and the HuH7 human hepatoma cell line. HuH7 cells are highly proliferating cells in which signaling pathways are strongly activated in response to extracellular stimuli [29, 30]. CRKL and FER expressions were silenced using 2 different siRNAs per targeted gene and progression through the G1/S phase was analyzed by EdU (5-ethynyl-2’-deoxyuridine) and/or methyl-^3^H thymidine incorporation in siRNA-inhibited *vs.* control cells. The efficiency of siRNA was validated by the 85% and 90% decrease in the expression levels of CRKL and FER in HeLa and HuH7 cell lines, respectively (Figure 7A and B). As shown in Figure 7C, D and E, CRKL and FER silencing induced a strong decrease in EdU and methyl-^3^H thymidine incorporation in both HeLa and HuH7 cells, highlighting a decrease in DNA replication. In addition, these effects were associated with a decrease in ERK phosphorylation (Additional file 1: Figure S3) and Ki67 expression (Additional file 1: Figure S4) thereby suggesting the implication of FER and CRKL in regulation of cell proliferation through mitogenic ERK-dependent pathways. To illustrate the cell cycle distribution with knockdown of CRKL and FER, we analyzed the cyclin D1 which plays a critical role in late G1 phase progression. We showed that cyclin D1 expression accumulated in CRKL and FER silenced cells while expression of CDK1 was not changed compared to controls. These data, together with methyl-thymidine inhibitions, support evidences for the implication of CRKL and FER in late G1 and G1/S transition (Additional file 1: Figure S5).

Interestingly we further observed a diminished migration of cells silenced for FER and CRKL (Additional file 1: Figure S6) that confirm and extend previous work showing that CRKL and FER could be associated with the metastatic potential of hepatocellular carcinoma (HCC) cells. Phosphoproteomic techniques based on LC-MS/MS and protein-protein interactions in crosstalk pathways first implicated FER in the invasive ability of metastatic hepatoma cells [26]. More recently, Liu et al. [31] demonstrated that CRKL could be a novel prognostic marker in HCC, whereby knockdown of CRKL in HCC cells leads to a decrease in cell migration and in the epithelial-mesenchymal transition process. In addition, high expression levels of CRKL and of the CRKL-FLT1 complex (a member of the vascular endothelial growth factor receptor family) strongly correlate with reduced disease-free and overall survival in HCC patients. Together these data demonstrate that targeting FER and CRKL might constitute a promising new therapeutic approach.

## Conclusions

Unlike previous RNAi-based screens, we have developed an original integrative data analysis to identify kinases required for cell proliferation. Using ontology annotation, we first identified two functional kinase groups differentially implicated in the control of cell proliferation by regulating either the cell cycle and cell division or, more broadly, signaling pathways. Second, we integrated signaling kinases with protein and phosphoproteomic interactions to generate a global view of kinase networks, including substrates and interacting proteins. Network analysis then allows for the identification of functional modules that regroup kinases working together. This is the case for FER and CRKL, which control mitogenic ERK-dependent signaling pathways.

Targeting specific signaling kinases that control cell proliferation has been developed as a strategy for cancer therapy, but resistance often emerges as a major impediment to effective chemotherapy. Systems biology approaches are necessary to take into account signaling complexity and to implement effective combinatorial therapies. The simple framework proposed in this work should serve as a useful basis to improve our understanding and interpretation of screening data and to facilitate the identification of new kinase functionalities that can in turn be used as part of new therapeutic strategies.

## Methods

### Cell culture and RNAi screening

Synthesized siRNA libraries were spotted onto 96 well microplates (200 pmole/well) by Sigma Aldrich (MISSION® siRNA Human Gene Family Panels). The panel contains over 2142 siRNA duplexes that target 714 kinase genes. 3 individual siRNA duplexes per target gene were designed using the Rosetta Inpharmatics design algorithm. siRNAs were solubilized in 25 μl serum-free OptiMem cell culture medium containing 0.3 μl DharmaFECT transfection reagents (Thermo Scientific Dharmacon®, Illkirch, France) for 20 min. HeLa cells were retrotransfected by plating 2.5×10^4^ cells per well in 175 μl of antibiotic-free Dulbecco’s modified Eagle’s medium supplemented with 10% fetal bovine serum. After a medium change, full medium was added 5 h post-transfection and cells were cultured for 48 h under 5% CO_2_ at 37°C. Plk1, a highly efficient inhibitor of cell proliferation and mitosis, was selected as an internal control in our study and it was spotted in all plates.

### Immunofluorescence and image acquisition

48 h post-transfection, cells were centrifuged and fixed with 8% formaldehyde for 20 minutes. The supernatants were discarded and the cells were washed twice with phosphate-buffered saline (PBS). Cells were permeabilized in PBS containing 0.1% Triton X-100 for 5 minutes. After washing with PBS, non-specific sites were blocked with 1% BSA for 15 minutes and cells were then incubated with monoclonal Anti-phospho-Histone H3 (Ser10) (Millipore, Molsheim, France) for 90 minutes at room temperature. Cells were washed twice with PBS, incubated with Alexa Fluor® 488 goat anti-mouse (In Vitrogen-Lifr technologies, CA, USA) for 30 minutes before staining DNA with DAPI (5 mg/ml for 5 minutes at room temperature). Color micrographs were taken using a Zeiss Axiolmager M1 equipped with a motorized stage (Märzhäuser Wetzlar). Images from the Zeiss ZVI files were converted into jpeg format and Cell Profiler software ( http://www.cellprofiler.org/) was used to perform automatic identification and measurement of biological objects. Briefly, the original color image was first converted to an image with varying grayscale intensities. Images were corrected for uneven illumination for each channel (DAPI and Alexa488 signals) and converted into binary images by grayscale Image Thresholding. Using the IdentifyPrimAutomatic module, regions of interest were then identified on the basis of size and fluorescent intensity. All parameters for each channel, DAPI for total cell number and Alexa488 for mitotic cells were exported and used for statistical analyses.

### siRNA transfection

Small interfering RNA (siRNA) oligonucleotides (Eurogentec, Belgium) were designed according to the manufacture’s recommendations. The following sequences with two 3’ deoxythymidine overhangs were used: siFER(d1), 5’-GAA AGA GCC ACC UCC AGU A-3’; siFER(d2),5’-GAA GGA AUU ACU AGA GCA A-3’ ; siCRKL (d1), 5’-CUC UCA UAG GCA AGU CAC A-3’; siCRKL (d2), 5’-GGA UGA AUA UAA AUG GCC A-3’; and a scramble control siRNA. Transfection of siRNAs was performed at 24 h of culture using Transfectin lipid reagent (BioRad, France). Cells cultured in 35-mm diameter plates were incubated with the transfection mix containing 100 nM siRNA and 5 μl of Transfectin in 1 ml of OptiMEM (Invitrogen, France), according to the manufacturer’s instructions. After 5 h of incubation, the transfection medium was removed and cells were switched to serum-free medium.

### EDU staining and cell proliferation assay

EdU (5-ethynyl-2’-deoxyuridine) directly measures de novo DNA synthesis or S-phase synthesis of the cell cycle using a method of covalently coupling an azide with an alkyne. Cells were cultured in DMEM medium supplemented with 5% FCS and treated with 1 μM EdU for three hours. After removal of supernatant, cells were washed with PBS and detection of EdU incorporation into the DNA was performed with the Click-iT1 EdU Alexa Fluor1 488 Cell Proliferation Assay Kit (Molecular Probes, Invitrogen, OR, USA) according to the manufacturer’s instructions.

### [3H] thymidine incorporation

The rate of DNA synthesis was also measured by adding 2 μCi of [methyl-3H] thymidine (5 Ci/mmol, Amersham Pharmacia Biotech) for given periods of time prior to cell harvesting and precipitation with ice-cold trichloroacetic acid (5%). Results are expressed as a percentage of control [methyl-3H] thymidine incorcoration.

### Migration assay

The scratch wound healing assay has been used to study the effects of CRKL and FER silencing on cell migration. Cells were grown in DMEM supplemented with 10% FBS. The confluent monolayer was scratched using a pipette tip and gently washed twice with medium to remove the detached cells. Cells were grown for additional 72 h and images were captured on a microscope.

### Western blot

Cells lysates were analyzed by sodium dodecyl sulfate-polyacrylamide gel electrophoresis and immunoblotting. Proteins were transferred electrophoretically to nitrocellulose membranes (Amersham, Buckinghmashire, UK). The blots were incubated for 1 hour in TBS containing 0.1% Tween 20 and 5% non-fat dry milk and further incubated for 1 hour with specific antibodies against CRKL and FER (Cell Signaling, Boston, USA). Bound antibodies were visualized with horseradish peroxidase-conjugated antibodies anti-rabbit or anti-mouse IgG (BioRad, Ivry, France) using an enhanced chemiluminescence system.

### Statistical analyses and normalization

Four independent images per well were analyzed with an average of 700 cells per image. Each 96-well plate contained internal controls including untreated cells, lipofectant-treated cells and siRNA Plk1-transfected cells as control for a positive inhibitor of the cell cycle. All parameters for each image were exported from CellProfiler. Data were pre-processed and analyzed using R and the CellHTS2 package [32], freely available on the Bioconductor project website ( http://www.bioconductor.org/). This package was especially developed to pre-process cell-based assays. Raw measurements were pre-processed with two non-specific filtering steps to remove unreliable measurements. First, measurements with DAPI intensities in the first percentile (lower bound) of the distribution of all DAPI intensities were removed. Second, all measurements where the estimated number of mitotic cells (Alexa488 signal) was higher than the total cell number (DAPI signal) were removed. Next, data were normalized within each channel: each measurement was divided by the plate median and magnified by the overall median (across plate). The mitotic index was calculated next as the ratio between the number of phosphohistone-positive cells and DAPI-positive cells Then, data were summarized and finally z-scores were estimated, according to the preprocessing work-flow for two-channel screens as described in http://bioconductor.org/packages/2.5/bioc/vignettes/cellHTS2/inst/doc/twoChannels.pdf). Kinases were selected for further analysis if their mitotic index was above or below the overall median of mitotic indexes plus or minus twice the median absolute deviation (median +/- 2MAD), respectively.

### Gene ontology and phospho-network analysis

Using Fatigo, a web interface which carries out simple data mining using gene ontology, we compared GO functional enrichment (based on a hypergeometric distribution) of kinases for which RNA interference increased (High) or decreased (Low) the mitotic index (MI) using a Fisher´s exact test ( http://babelomics3.bioinfo.cipf.es/) [33]. Di-acyclic graphs for cellular component ontology were generated using the Gene Ontology Tree Machine (GOTM, http://bioinfo.vanderbilt.edu/webgestalt/) [34].

The kinase interactome networks were built using PhosphoPOINT, the human interactome and phosphoprotein database ( http://kinase.bioinformatics.tw/) [35]. All proteins interacting with identified kinases were extracted from the PhosphoPOINT database and parsed into a CVS file. The Cytoscape graph editor was used to visualize networks ( http://www.cytoscape.org/index.php) [36] and ClueGO: a Cytoscape plug-in was used to decipher functionally grouped gene ontology and pathway annotation networks [37].

## Electronic supplementary material

Additional file 1: **The following additional data are available with the online version of this paper.** **Table S1.** Lists protein kinases screened and their associated siRNA sequences. **Table S2.** Describes row data of MI for the 714 siRNAs. **Table S3.** Compares identified kinases with results from the literature. **Table S4.** Describes protein-protein interaction network from the low-MI group. **Table S5.** Describes protein-protein interaction network from the high-MI group. **Table S6.** Describes parameters of topological analyses from low and high MI graphs. **Figure S1 and S2.** Are expandable views of PPIs from high- and low-MI groups, respectively. **Figure S3.** Describes the effects of FER and CRKL silencing in HuH7 cells on ERK phosphorylation. **Figure S4.** Shows KI67 expression in HuH7 cells silenced for FER and CRKL. **Figure S5 and** **Figure S6.** Describes the effect of FER and CRKL silencing in HuH7 cells on Cdk2 expression and migration, respectively. (ZIP 910 KB)

## Abbreviations

EdU: 5-ethynyl-2’-deoxyuridine
FER: FPS/FES related tyrosine kinase
CRKL: CRK-like: v-crk, avian sarcoma virus CT10 oncogene homolog-like
HCC: Hepatocellular carcinoma
PI: Propidium iodide
RNAi: RNA interference
siRNA: Small interfering RNA
shRNA: Small hairpin RNA
PPI: Protein-protein interaction.
