# Supplementary figures and images for: Integrative analysis of high-throughput RNAi screen data identifies the FER and CRKL tyrosine kinases as new regulators of the mitogenic ERK-dependent pathways in transformed cells
Source: BMC Genomics. 2014 Dec 23;15(1):1169. doi: 10.1186/1471-2164-15-1169 (PMC4367906; doi:10.1186/1471-2164-15-1169)

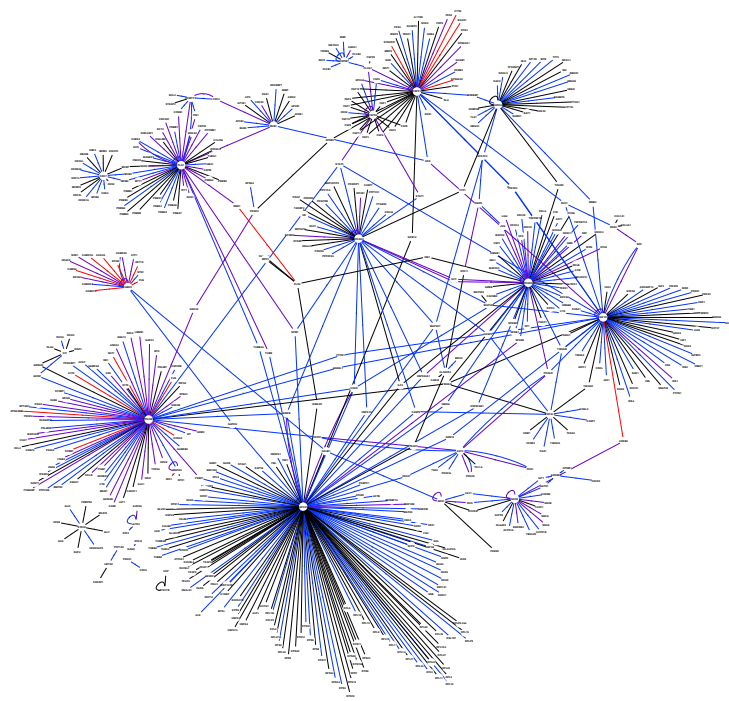

Supplement: Supplementary file 1 — Additional file 1: The following additional data are available with the online version of this paper. Table S1. Lists protein kinases screened and their associated siRNA sequences. Table S2. Describes row data of MI for the 714 siRNAs. Table S3. Compares identified kinases with results from the literature. Table S4. Describes protein-protein interaction network from the low-MI group. Table S5. Describes protein-protein interaction network from the high-MI group. Table S6. Describes parameters of topological analyses from low and high MI graphs. Figure S1 and S2. Are expandable views of PPIs from high- and low-MI groups, respectively. Figure S3. Describes the effects of FER and CRKL silencing in HuH7 cells on ERK phosphorylation. Figure S4. Shows KI67 expression in HuH7 cells silenced for FER and CRKL. Figure S5 and Figure S6. Describes the effect of FER and CRKL silencing in HuH7 cells on Cdk2 expression and migration, respectively. (ZIP 910 KB) [file 12864_2014_6952_MOESM1_ESM.zip › 1533173142128948_add1.pdf]

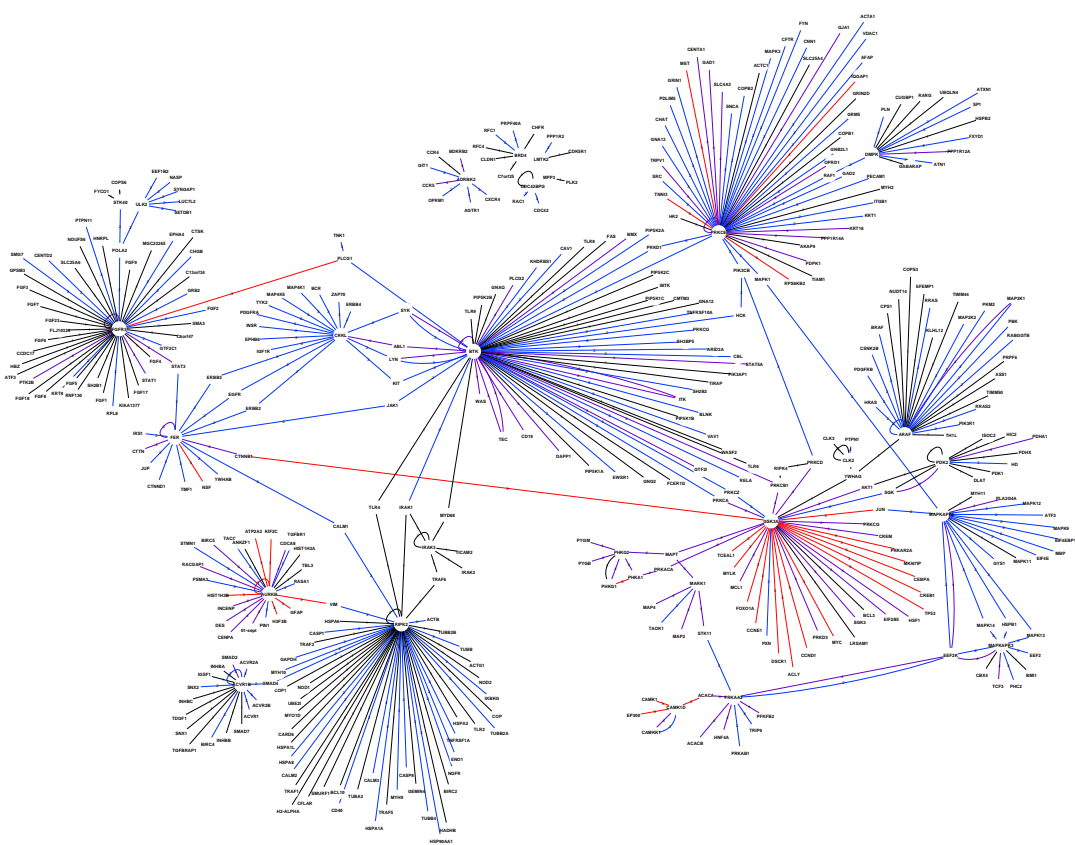

Supplement: Supplementary file 1 — Additional file 1: The following additional data are available with the online version of this paper. Table S1. Lists protein kinases screened and their associated siRNA sequences. Table S2. Describes row data of MI for the 714 siRNAs. Table S3. Compares identified kinases with results from the literature. Table S4. Describes protein-protein interaction network from the low-MI group. Table S5. Describes protein-protein interaction network from the high-MI group. Table S6. Describes parameters of topological analyses from low and high MI graphs. Figure S1 and S2. Are expandable views of PPIs from high- and low-MI groups, respectively. Figure S3. Describes the effects of FER and CRKL silencing in HuH7 cells on ERK phosphorylation. Figure S4. Shows KI67 expression in HuH7 cells silenced for FER and CRKL. Figure S5 and Figure S6. Describes the effect of FER and CRKL silencing in HuH7 cells on Cdk2 expression and migration, respectively. (ZIP 910 KB) [file 12864_2014_6952_MOESM1_ESM.zip › 1533173142128948_add2.pdf]
